# Chitosan Biomaterials: Advances and Challenges

**DOI:** 10.3390/ijms242216150

**Published:** 2023-11-10

**Authors:** Lăcrămioara Popa, Mihaela Violeta Ghica, Cristina-Elena Dinu-Pîrvu

**Affiliations:** Department of Physical and Colloidal Chemistry, Faculty of Pharmacy, University of Medicine and Pharmacy “Carol Davila”, 020956 Bucharest, Romania; lacramioara.popa@umfcd.ro (L.P.); cristina.dinu@umfcd.ro (C.-E.D.-P.)

The purpose of this Special Issue was to review research focusing on the development of formulations based on chitosan or its derivatives together with other molecules, producing biomaterials with improved physicochemical properties and effects. The lack of drinking water, especially in disadvantaged countries, is an increasingly discussed topic and represents a concern at the level of the World Health Organization. One of the manuscripts is based on this topic and presents a water treatment method designed to ensure access to drinking water. The theme of the other manuscripts mainly concerns the development of systems based on a chitosan biopolymer, but they also discuss the preparation, evaluation, and effects of these systems in the bio-medical field.

The applicability of chitosan in the medical field (drug delivery systems and tissue engineering) and other fields (food, cosmetics, and agriculture) is due to its already-well-known properties. Chitosan is a natural polymer, obtained via the deacetylation of chitin. It is biodegradable, biocompatible [1,2], bioabsorbable, and non-toxic [3].

Concerns regarding the world population’s access to drinking water have increased in recent years, and have led to new water treatment and filtration procedures. The method developed by Holmes and his team combined chitosan’s ability to coagulate substances suspended in water, which subsequently flocculate, with the technique of water filtration through sand columns, thus removing the floaters that contain impurities in water, including bacteria and viruses. Chitosan concentrations of 3, 10, and 30 mg/L were used to pretreat the water. The highest retained amount of bacteria and viruses was recorded at the concentrations of 10 mg/L and 30 mg/L of chitosan. The best result in terms of turbidity was in the case of water pretreated with 10 mg/L chitosan followed by filtration using sand columns [4].

The properties of chitosan and its derivatives, which help to enhance drug permeation through the skin, have led to the development of pharmaceutical systems in association with other natural or synthetic molecules, such as films, scaffolds, membranes, and nanoparticles. The results of the evaluation of these systems have shown that they are hemocompatible, biocompatible, and do not cause skin irritation [5]. A chitosan biopolymer’s main asset is its mucoadhesive property, because this increases the residence time of formulations at the oral, nasal, vaginal, or skin level, and thus the bioavailability of the active pharmaceutical ingredient is improved [6].

In the case of wound treatment, in addition to the mucoadhesive property, hemostatic and antimicrobial actions play an important role in the healing process. Chitosan is generally used together with other biomaterials, obtaining smart-materials that stimulate wound healing [5,7].

The antimicrobial action of chitosan and its derivatives can be increased through association with other molecules or plant extracts, and this can help to obtain a synergy between the properties of chitosan and plants with similar applicability in several therapeutic areas [8].

The study carried out by Azueta-Aguayo, P.H. and collaborators on scaffolds based on chitosan and ammonium hydroxide, for use in the field of tissue engineering, showed that their structures are sufficiently porous and can help to increase cell development. Regarding formulations based on chitosan and neutralized ammonium hydroxides, the thermal stability and elastic properties were superior. Furthermore, for the non-neutralized scaffolds, the direct cytotoxicity and proliferation tests showed that a greater number of cells adhered to the scaffolds and their viability increased after 48 h. In conclusion, scaffolds based on chitosan and neutralized ammonium hydroxide have the potential to be used in the medical field due to their non-toxic and biocompatible properties [3].

Peng and colleagues developed and evaluated chitosan-coated oxymatrine liposomes for inhalation administration based on the antiviral action of oxymatrine, especially regarding the respiratory syncytial virus (RSV). Due to the mucoadhesive properties of chitosan, the retention time at the lung level of liposomes covered with chitosan is higher, meaning an improved bioavailability of oxymatrine. Preliminary in vitro experiments showed that liposomes with oxymatrine coated with chitosan inhibited the proliferation of lethal RSV induced in mice. The research results demonstrated that chitosan-coated liposomes have the potential to be used as inhalation drug delivery systems, but studies are needed to optimize them [9].

A challenging topic studied lately has been the intranasal administration of drugs, due to its advantages. Many drug administration systems are based on chitosan or its derivatives, such as gels for the intranasal administration of antihistamines (loratadine and chlorpheniramine) [10]; insulin [11]; liposomes and nanoemulsions for the administration of a derivative of Dehydroepiandrosterone (DHEA) [12]; microemulsion with silymarin for the treatment of Parkinson’s disease [13]; nanoparticles with galantamine [14] or ropinirole [15]; and nanocrystals with memantine for the treatment of Alzheimer’s disease [16].

In this Special Issue, ”Chitosan Biomaterials: Advances and Challenges”, the published articles offer a significant contribution in studying the properties of chitosan as a biomaterial and are of interest to those researching the development and optimization of chitosan-based systems. Perspectives for the use of chitosan are still very broad, and its applications are still in the initial stages of development.
